# Exercise-induced bronchoconstriction, allergy and sports in children

**DOI:** 10.1186/s13052-024-01594-0

**Published:** 2024-03-13

**Authors:** Angela Klain, Mattia Giovannini, Luca Pecoraro, Simona Barni, Francesca Mori, Lucia Liotti, Carla Mastrorilli, Francesca Saretta, Riccardo Castagnoli, Stefania Arasi, Lucia Caminiti, Mariannita Gelsomino, Cristiana Indolfi, Michele Miraglia del Giudice, Elio Novembre

**Affiliations:** 1 Department of Woman, Child and General and Specialized Surgery, University of Campania Luigi Vanvitelli, 80138 Naples, Italy; 2 Allergy Unit, Meyer Children’s Hospital IRCCS, 50139 Florence, Italy; 3 Department of Health Sciences, University of Florence, 50139 Florence, Italy; 4 Pediatric Unit, Department of Surgical Sciences, Dentistry, Gynecology and Pediatrics, University of Verona, 37126 Verona, Italy; 5 Pediatric Unit, Department of Mother and Child Health, Salesi Children’s Hospital, 60123 Ancona, Italy; 6 Pediatric and Emergency Department, Pediatric Hospital Giovanni XXIII, AOU Policlinic of Bari, 70126 Bari, Italy; 7 Pediatric Department, Latisana-Palmanova Hospital, Azienda Sanitaria Universitaria Friuli Centrale, 33100 Udine, Italy; 8 Department of Clinical, Surgical, Diagnostic and Pediatric Sciences, University of Pavia, 27100 Pavia, Italy; 9 Pediatric Clinic, Fondazione IRCCS Policlinico San Matteo, 27100 Pavia, Italy; 10 Translational Research in Pediatric Specialties Area, Division of Allergy, Bambino Gesù Children’s Hospital, IRCCS, 00165 Rome, Italy; 11 Allergy Unit, Department of Pediatrics, AOU Policlinico Gaetano Martino, 98124 Messina, Italy; 12 Department of Life Sciences and Public Health, Pediatric Allergy Unit, University Foundation Policlinico Gemelli IRCCS, Catholic University of the Sacred Heart, 00168 Rome, Italy

**Keywords:** Exercise-induced bronchoconstriction, Exercise-induced asthma, Children, Sport, Atopy, Allergy, Asthma, Diagnosis, Treatment

## Abstract

Exercise-induced bronchoconstriction (EIB) is characterized by the narrowing of airways during or after physical activity, leading to symptoms such as wheezing, coughing, and shortness of breath. Distinguishing between EIB and exercise-induced asthma (EIA) is essential, given their divergent therapeutic and prognostic considerations. EIB has been increasingly recognized as a significant concern in pediatric athletes. Moreover, studies indicate a noteworthy prevalence of EIB in children with atopic predispositions, unveiling a potential link between allergic sensitivities and exercise-induced respiratory symptoms, underpinned by an inflammatory reaction caused by mechanical, environmental, and genetic factors. Holistic management of EIB in children necessitates a correct diagnosis and a combination of pharmacological and non-pharmacological interventions. This review delves into the latest evidence concerning EIB in the pediatric population, exploring its associations with atopy and sports, and emphasizing the appropriate diagnostic and therapeutic approaches by highlighting various clinical scenarios.

Exercise is one of the most important triggers of acute asthma attacks. The concept of exercise-induced asthma (EIA) has been introduced almost 60 years ago [1]. Previously, EIA enclosed all the respiratory conditions associated with exercise, even transient bronchoconstriction. Exercise incorrectly represented the cause and the trigger of asthma attacks because most asthmatic patients also have bronchoconstriction. Afterwards, the term exercise-induced bronchoconstriction (EIB) was introduced, but EIA and EIB have been incorrectly used interchangeably [2]. Finally, during the first decade of 2000s, a consensus between the Joint Task Force on Practice Parameters (JTFPP), the American Academy of Allergy, Asthma & Immunology (AAAAI), the American College of Allergy, Asthma & Immunology (ACAAI), the Joint Council of Allergy Asthma & Immunology (JCAAI), the Joint Task Force of the European Respiratory Society (ERS) and the European Academy of Allergy and Clinical Immunology (EAACI) has ‘officially’ differentiated the two conditions, recommending naming EIB with asthma (EIBa), the occurrence of bronchial obstruction during exercise in asthmatic patients, and EIB without asthma (EIBwa), the occurrence of EIB in individuals without other signs and symptoms and indicators of asthma (3–4).

EIA defines, therefore, a clinical condition of bronchial inflammation in which exercise is one of the main trigger factors and which overlaps and exacerbates a pre-existing underlying condition [5].

EIB is defined as acute airway narrowing (transient and reversible) that occurs during or after exercise and can be observed in patients with chronic asthma and those without [6]. EIB clinical manifestations include shortness of breath, wheezing, cough, decreased endurance, and tightness in the chest [7]. EIB reportedly usually occurs within 2 − 5 min after exercise, peaks after 10 min, and resolves in approximately 60 min.

The prevalence of EIB varies from 5 to 20% in the general population [8]. In children ≤ 16 years old, the prevalence of EIB is also higher than in the general population, ranging from 3 to 35%, with a considerable variation in the prevalence of EIB in children worldwide due, e.g., to ethnicity, urban-rural, and socioeconomic differences [9]. In Caucasian inner-city children, the prevalence is 4.5% [10]. EIBa occurs more frequently in severe and uncontrolled asthmatic patients [3]. EIBwa is common in athletes, children, subjects with rhinitis, and following respiratory infections [7, 11, 12].

## Atopy and EIB

Atopy and EIB are closely related. Atopy is one of the most relevant risk factors in the development of EIB in children, as epidemiological evidence demonstrates that up to 40% of infants with EIB have allergic rhinitis (AR), and 30% of them may acquire asthma in adulthood [5, 13–16]. EIB is seen in 40–90% of asthmatic kids, particularly in those with severe uncontrolled asthma [3, 17, 18].

The degree of asthma severity was associated with the likelihood of experiencing exercise-induced wheezing. Severe asthmatic patients have a significantly higher risk of exercise-induced wheezing, with a 4 to 6-fold increase compared to individuals who suffer from intermittent asthma attacks [19].

Finally, the exercise itself can induce respiratory, systemic, and skin reactions [20–22]. Exercise-induced rhinitis is frequent, especially in outdoor sports such as winter athletes [23]. About 20–40% of children with rhinitis have EIB, particularly those with persistent untreated signs and symptoms [23, 24]. On this basis, the Allergic Rhinitis and its Impact on Asthma (ARIA) guidelines recommend screening every subject with rhinitis, including athletes, for asthmatic clinical manifestations [25]. Moreover, rhinitis alone and rhinitis and asthma multimorbidity seem to represent two distinct diseases with different courses [26].

Exercise-induced anaphylaxis is a rare condition in which anaphylaxis occurs after physical activity. The signs and symptoms may include pruritus, hives, flushing, dyspnoea, wheezing, and gastrointestinal clinical manifestations, including nausea and diarrhoea. If strenuous activity is continued, patients may develop more severe signs and symptoms, such as angioedema, laryngeal oedema, hypotension, and cardiovascular collapse [21].

The differential diagnosis of EIB should consider these conditions and other diseases leading to shortness of breath during exercise, such as exercise-induced laryngospasm, exercise-induced anaphylaxis, cardiovascular, pulmonary, or gastrointestinal, and metabolic illnesses [27].

## Predictors of EIB

Numerous factors associated with EIB have been discovered, and the presence of multiple risk elements may increase the likelihood of developing this condition.

In the research by Lin et al., investigating the prevalence and predictors of EIB in children with asthma, EIB occurred in 52.5% of asthmatic children: children with EIB suffered more from atopic dermatitis (*p* = 0.038) and allergy to *Dermatophagoides pteronyssinus* and *Dermatophagoides farinae* than non-EIB patients (*p* = 0.045 and 0.048 respectively). Patients, who were more sensitive to methacholine challenge (with lower PC_20_ levels), developed EIB with more decline in FEV_1_ after exercise challenge (*p* = 0.038) [28].

In a primary school, a study was conducted within a community and involved children aged 6 to 12 years. Peak Expiratory Flow Rate (PEFR) measurements were taken both at rest and after a 6-minute free running test conducted on the school playground, utilizing a peak flow meter. A diagnosis of EIB was established if there was a PEFR decline of at least 10%. Subsequently, those who exhibited EIB were further classified based on the extent of PEFR decline post-exercise: a decline of ≥ 10% but less than 25% was categorized as mild EIB, ≥ 25% but less than 50% as moderate EIB, and ≥ 50% as severe EIB. These groups were then collectively referred to as individuals with either EIBA or EIBwa, depending on their asthma status. The results showed that 58% of the children with EIB were of high social class, and most children with EIB had EIBwa (84.1% vs. 15.9% of EIBa). History of AR and atopic dermatitis were more frequent in children diagnosed with EIB (OR5.832, *p* = 0.001; OR2.740, *p* = 0.003, respectively) [14].

In a retrospective study, Park et al. reviewed electronic medical records of patients who visited an allergy clinic for respiratory signs and symptoms after exercise and underwent exercise bronchial provocation testing. Patients older than 18 years of age presenting with respiratory clinical manifestations while exercising who underwent exercise provocation testing were included in the study. EIB was observed in 66.9% of the subjects, and sputum eosinophilia was more frequent in EIB patients than in non-EIB patients (*p* = 0.037). The temperature and relative humidity on exercise test day were significantly related to the EIB-positive rate. Airflow limitation development in methacholine-positive EIB patients was relatively more abrupt and severe compared with methacholine-negative EIB patients [29]. Total IgEs, atopy rate, and house dust mite sensitization rate did not differ between EIB-positive and negative patients.

In the study by Martín-Muñoz et al., children between 6 and 14 years old were included: 31% had experienced coughing or wheezing during exercise for at least three months and also tested positive on the exercise challenge test (ECT). Among these children, 22% had only EIB (referred to as group A), while 78% (group B) had asthma with EIB. Additionally, 41 children with controlled asthma (group C) showed good tolerance for exercise and negative ECT results. The analysis of the EIB patients (groups A, B) versus group C demonstrated a clear association between EIB suffering and sensitization to indoor allergens (*p* = 0.023); OR 6.3 [30].

Fractional exhaled nitric oxide (FeNO) represents an indicator of airway inflammation in asthma, and elevated FeNO levels can predict the subsequent development of asthma in otherwise healthy children [31]. In the study by Grzelewski et al., elevated FeNO levels independently raised the likelihood of EIB in schoolchildren with asthma, regardless of other indicators of asthma severity and the effectiveness of anti-asthma treatments [32]. Based on Buchvald et al. research, the likelihood of excluding EIB reaches 90% when the FeNO50 levels are below 20 parts per billion (p.p.b.) in individuals who are not presently utilizing inhaled corticosteroids and below 12 p.p.b. in those who are currently using inhaled corticosteroids [33]. Therefore, FeNO has been proposed as a valuable tool to predict EIB in asthmatic children [32–35].

## Underlying mechanisms of EIA and EIB

**Fig. 1:**
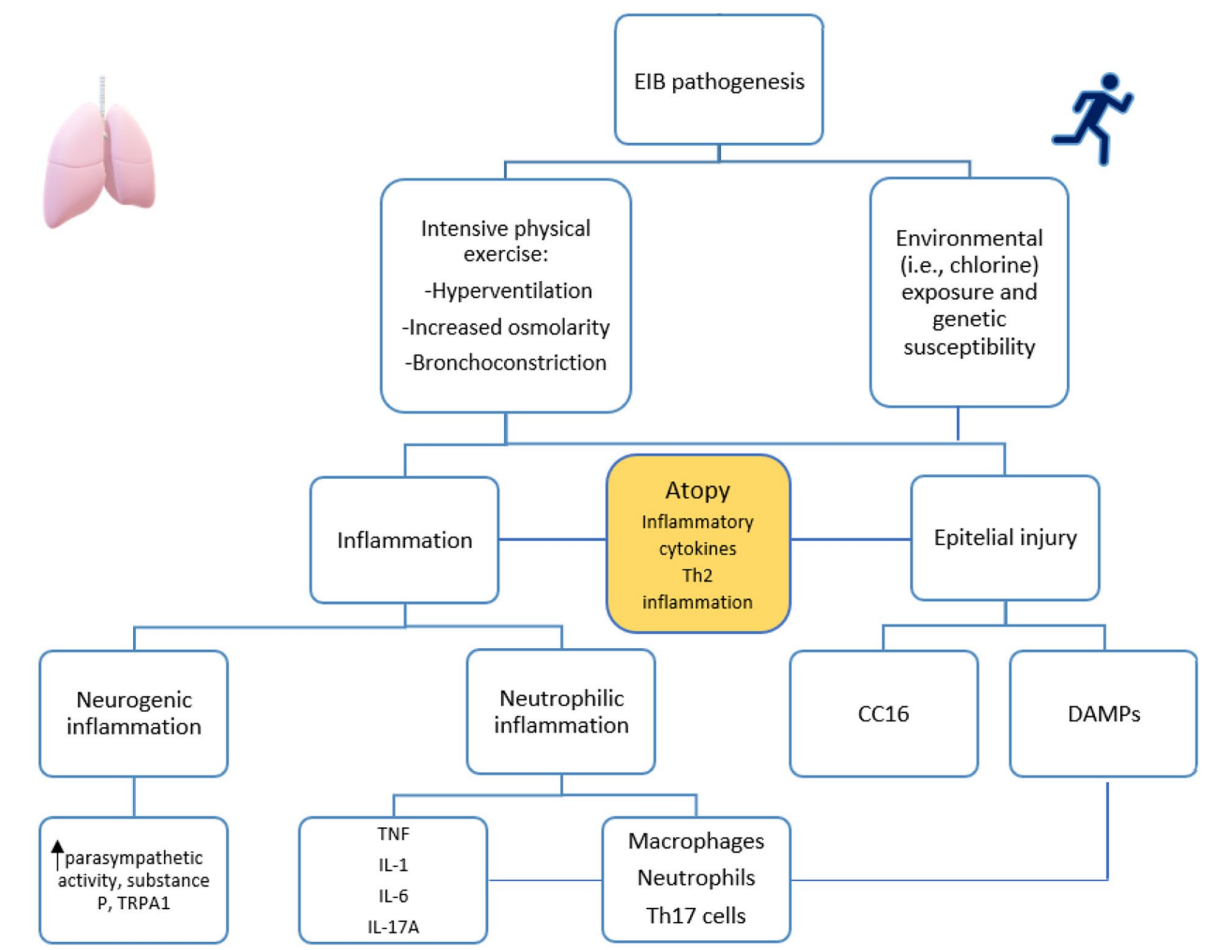
EIB pathogenesis and the central role of atopy EIB: exercise-bronchoconstriction, Th2: T helper cell type 2, TRPA1: transient transient receptor potential ankyrin 1 channel receptor potential ankyrin 1 channel, TNF: Tumor necrosis factor, IL-1: interleukin 1, IL-6 interleukin 6, IL-17 A: interleukin 17 A, Th17: T helper cell 17, CC16: Clara cell secretory protein, DAMPs: damage-associated molecular patterns

Specifically, it is believed that the development of the condition is underpinned by cellular and neurogenic inflammation, as well as cellular damage, triggering a series of events that lead to bronchoconstriction (Fig. 1). These phenomena become especially noticeable in individuals exposed to irritants associated with certain sports, such as chlorine for swimmers, pollution for runners or cyclists, cold and dry air for ice skaters or hockey players, as well as exposure to perfumes, cleaning agents, paint, new equipment, or carpets in gyms [8, 36].

Intense physical activity leads to the intake of larger amounts of relatively cold and dry air, which triggers significant pathogenic processes associated with EIA and EIB. These processes include alterations in airway osmolarity, epithelial damage, airway inflammation, and activation of nerve cells. This initial hypothesis is called the osmotic hypothesis: exercise increases ventilation, leading to mouth breathing, cooling and dehydration of the airway mucosa [24]. As a result of dehydration, liquid layer osmolarity increases, causing the release of mediators, including prostaglandins, leukotrienes, and histamine, leading to smooth muscle contraction and altering vascular permeability [37]. The cooling of the airways triggers the activation of cholinergic receptors in the airways, leading to an elevation in airway smooth muscle tone and the production of airway secretions [38]. In addition, the loss of airway surface water reduces mucociliary clearance acutely during hyperpnea of dry air [39]. During moderate-high exertion, the water supply in the major airways is insufficient to meet the demand for humidification, necessitating the recruitment of small airways. Compared to the first ten generations, these smaller airways have a significantly higher volume of surface fluids. Still, since they have a diameter of less than 1 mm, they are more susceptible to dehydration injury [40]. The degree of airway constriction for an individual, whether asthma is present or not, is likely to be influenced by their recruitment into the conditioning process of small airways [41]. Ending the exercise session would lead to vascular engorgement and oedema due to the rapid restoration of blood volume, resulting in epithelial injury and EIB. The process of repair may contribute to hyperemia and the development of airway hyperresponsiveness in healthy subjects.

New findings suggest that injury to the airway epithelium, as a consequence of intense exercise, plays a significant role in the susceptibility to EIB. Recently, it was shown that athletes exhibit increased levels of damage-associated molecular patterns (DAMPs), as acid uric, in their sputum, which are capable of activating immunity cells, such as macrophages, prompting the release of pro-inflammatory cytokines like TNF, IL-1b, or IL-6 [42]. These cytokines promote the differentiation of Th17 cells, which produce IL-17 A that induces neutrophil recruitment via the production of IL-8 by airway epithelial.

Clara cell secretory protein 16 (CC16) is a peripheral marker for evaluating the integrity of the epithelial barrier in the lower airways. Previous studies have demonstrated that acute exercise induces epithelial stress, as evidenced by increased levels of CC16 in urine, serum, and among athletes after eucapnic voluntary hyperventilation [43–45].

High-intensity and prolonged physical activity may also induce autonomic dysregulation. Parasympathetic activity, which induces bronchoconstriction, is increased in endurance-trained individuals compared to untrained counterparts [46]. Dysfunctional neuro-immune interaction may also play a role in the pathogenesis of EIB, involving a neurogenic inflammation course [46]. Increased levels of substance P, a major initiator of neurogenic inflammation, have been observed post-strenuous exercise [47]. Studies have shown that substance P-induced bronchoconstriction may be attributed to cholinergic activation, potentially via the transient receptor potential ankyrin 1 channel (TRPA1) [48]. Additionally, acetylcholine released from the respiratory epithelium may potentially stimulate airway inflammation [49]. Moreover, intense and prolonged physical exercise is linked to elevated serum levels of nerve growth factor in athletes [50].

Atopy significantly contributes to and amplifies these phenomena. Patients with AR suffer from runny nose nasal congestion, and pre-existing mouth breathing. These signs and symptoms during exercise are exacerbated, causing shortness of breath. Furthermore, epithelial injury, favored by the release of epithelial cytokines (or alarmins) after pollen-driven activation, is already evident in asthmatic patients. These processes collectively contribute to impaired mucociliary clearance and airway remodelling [51]. In asthmatic subjects, where persistent bronchial inflammation is the principal underlying mechanism [52, 53], acute exercise can exacerbate airway inflammation: hyperventilation induces epithelial damage and increases the production of leukotrienes and inflammatory cytokines (e.g., IL-1, TNF-alpha, IL-6), as evidenced in the bronchoalveolar lavage (BAL) of athletes with EIB [54, 55]. Furthermore, vascular factors such as Vascular Endothelial Growth Factor (VEGF) may also play a role in airway remodelling [56]. In addition, intense physical training may induce a transient status of immune downregulation with a shift toward a prevalent T-lymphocyte helper-2 response, clinically associated with an increased prevalence of atopy and viral upper respiratory tract infections, both representing relevant risk factors for the onset and worsening of asthma [56].

Finally, several genetic variations have been supposed in EIB pathogenesis, such as PPT-1 gene, responsible for generating substance P, aqueous water channel aquaporin (AQP5) gene, which forms the AQP 5, potentially contributing to the safeguarding of airway reactivity to nonspecific stimuli and CC16 gene [57, 58]. However, to date, the association between genetic alterations and the development of EIB has not been conclusively demonstrated, the need for further exploration is apparent.

Exercise may also negatively impact several aspects of asthmatic children’s daily lives, including school attendance, reduced participation in physical education classes, and, generally, their quality of life [59].

## EIB and sport

EIB is particularly frequent in athletes [54]. Atopy and training styles are independent risk factors for EIA/EIB: atopic power athletes have a 25-fold greater risk compared to non-atopic populations, long-distance runners have a 42-fold increase, and swimmers have a 92-fold increase [60]. In a systematic review, the prevalence of EIB among adolescent athletes with asthma ranged from 2.1 to 61%. This heterogeneity may be attributable, e.g., to the variable sample sizes, ethnic factors, environmental factors, and nature of study designs [61].

Nevertheless, EIB in athletes is still underdiagnosed both because many subjects, especially adolescents, hide their clinical manifestations and because self-reported exercise-induced dyspnea alone is a weak indicator for EIB [60, 62]. In athletes, EIB’s underlying mechanism would be found in repeated insults to the respiratory epithelium, caused by increased ventilation and dehydration, together with exposure to environmental factors such as cold air or chlorine in swimming pools. Repeated epithelial damage has the potential to decrease prostaglandin E2 and expose the underlying smooth muscle of the airway to plasma-derived substances that can change the muscle’s ability to contract. Paradoxically, plasma exudation may operate to increase airway hyperresponsiveness to exercise and other trigger stimuli [57].

In swimmers, many studies underline that the high prevalence of EIB is associated with repeated exposure to chlorinated pool water [63, 64]. On the contrary, in the meta-analysis by Valeriani et al., including seven reports, authors found no significant difference in asthma development between children attending swimming pools and controls (OR, 1.084; 95% CI: 0.89–1.31) [65]. In the Turkish article investigating the presence of EIB in adolescent elite swimmers, the authors observed that the overall prevalence of EIB(8.5%) was not different from that of the general population; furthermore, swimming exercise significantly increased FVC of swimmers [66]. The role of environmental conditions (chlorinated pool water) experienced by swimmers has been better explored in the study by Leahy et al. [67]. Collegiate swimmers (21 ± 2 year) completed three days of testing in pseudorandom order; a standard eucapnic voluntary hyperventilation test (EVH), a modified EVH test in the chlorinated environment (EVHCl), and a swimming test (swim). Spirometry was measured at baseline, and 3, 5, 10, 15, and 20 min after each test. Forced expired flow between 25% and 75% lung volume and peak expired flow were significantly reduced by the EVH compared with the EVHCl and swim tests (*P* < 0.05). The authors concluded that EVH elicits a greater FEV_1_ fall index compared with EVHCl, and swim and pool swimmers are potentially protected against bronchoconstriction.

Therefore, the chlorinated pool hypothesis is still controversial. Longitudinal studies are needed to definitely clarify any role of chlorinated swimming pool attendance in the development of asthma.

Several researchers, instead, highlight that the epithelium damage may be facilitated by an existent sensitization to aeroallergens or increase the risk of sensitization in non-atopic children by reducing the protective properties of the pulmonary epithelium [68]. A Belgian study reported an increased risk of childhood asthma in atopic swimmers with a total IgE of > 100 kIU/L [67]. Andersson et al., found an increased risk of asthma in sensitized children currently attending indoor swimming pools once a week or more [69].

Regular moderate exercise improves asthma control and quality of life [70]. This observation is confirmed by studies on the murine model of allergic asthma: low-to-moderate intensity regular aerobic exercise decreases eosinophilic and lymphocytic inflammation), airway remodelling, pro-inflammatory cytokine production (with a down-regulation of inflammatory mediators’ genes expression), FeNO levels, enhancing regulatory T cell (Treg) response (71–72). Moreover, regular exercise induces immunological changes, switching to a Th1 rather than Th2 response and decreasing total and allergen specific IgE levels in asthmatic patients [73]. In addition, a Cochrane-based meta-analysis of eight training trials, including asthmatic patients from 6 years of age, found that asthmatic adolescents and children who engage in systematic physical exercise become fitter and have a higher quality of life [74]. Thus, it is clear that aerobic, moderate-intensity exercise training (such as running or cycling) can be helpful for allergic inflammation. Exercise therapy for asthmatic patients, in which exercise, which is typically thought to be a potential trigger for EIA/EIB, is instead an integral part of the prevention and therapy strategies. Thereby, Global Initiative on Asthma (GINA) report recommend regular exercise for asthmatic children and adolescents [7].

## Diagnosis

Indirect stimulation tests, such as ECT, hypertonic saline or mannitol powder inhalation test, and EVH, may stimulate inflammatory cells in the respiratory tract, release chemical mediators such as histamine and leukotrienes, and cause the smooth muscle in the airway to contract. These tests are considered reasonable for the diagnosis of EIB, and EVH is regarded as the gold standard for this purpose in athletes.

ECT is generally practiced on a moving carpet with free running; The response to the test is considered positive when there is a fall in post-exertion FEV1 greater than or equal to 10% compared to baseline FEV1. Typically, bronchoconstriction begins immediately after exercise, reaches its maximum after 5–8 min and disappears spontaneously within 60 min [6, 75].

Hypertonic saline and mannitol powder inhalation tests, also known as hyperosmolar inhalation tests, lead to the contraction of smooth muscles in the airway through a mechanism that involves the evaporation of water and dehydration in the airways. The hypertonic saline provocation test can be conducted using 4.5% saline through an ultrasonic nebulizer with an output of 1 mL/mL. During the mannitol powder inhalation test, the concentration of inhaled mannitol powder is gradually increased, and the FEV1 is measured after each increment to evaluate the extent of airway contraction. In both provocation tests, a decrease in FEV1 of over 15% following inhalation is considered a positive result [76].

**Fig. 2:**
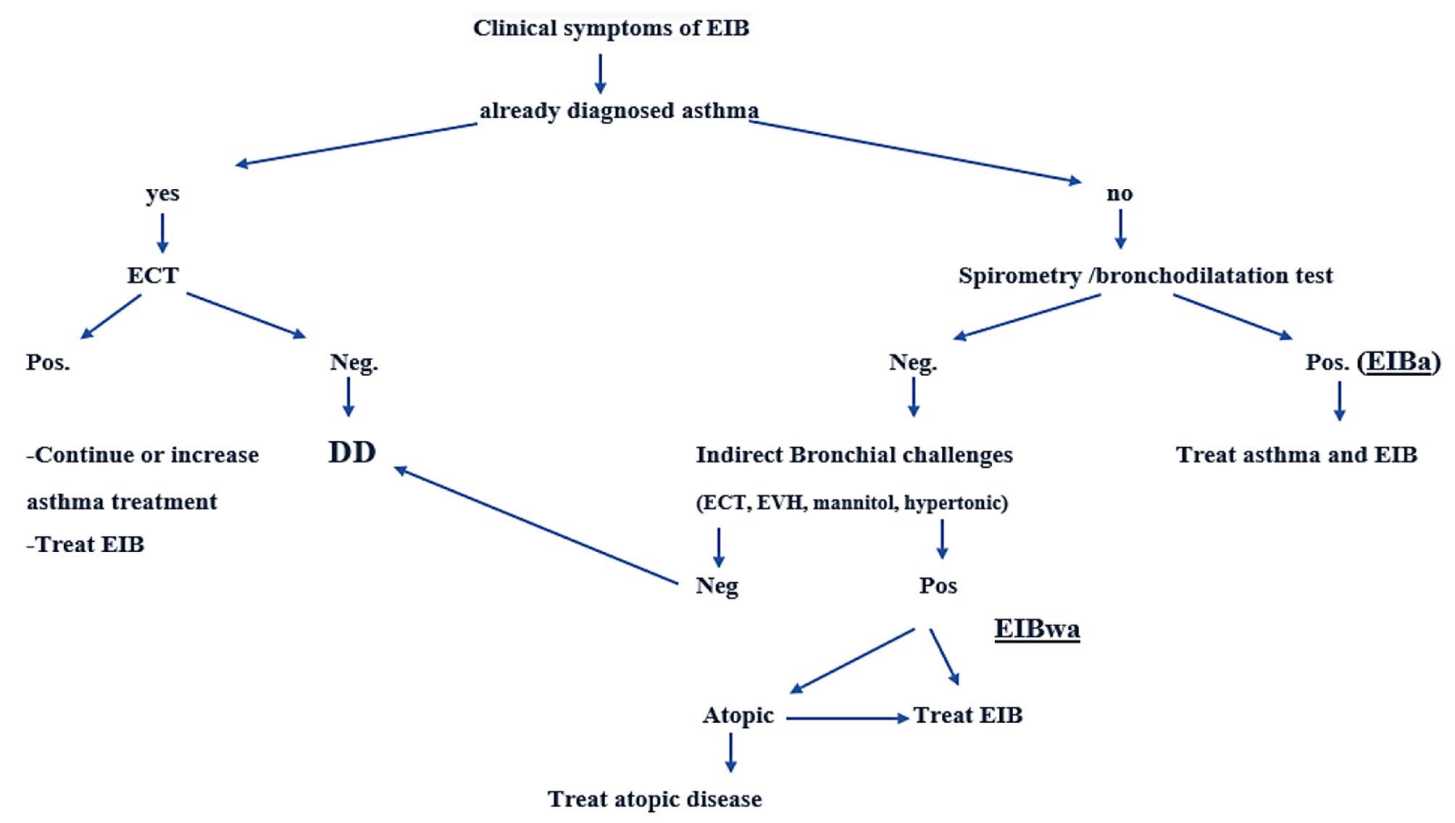
Algorithm for the diagnosis, treatment, and differential diagnosis of EIB. Adaptation from *John M. Weiler, J*. *Allergy Clin. Immunol.2016* [27] and *Klain et al. Front. Med,2022* [5]

EIB: exercise-induced bronchoconstriction; EIBwa: exercise-induced bronchoconstriction without asthma; EIBa: exercise-induced bronchoconstriction with asthma; ECT: exercise challenge test; EVH: eucapnic voluntary hyperpnoea; DD: differential diagnosis.

Another provocation test is the EVH, which is a medical test that involves controlled, rapid breathing to induce bronchoconstriction or airway narrowing in individuals suspected of having EIB. During an EVH test, the patient breathes in a specific gas mixture, usually dry, cool air with a lower concentration of carbon dioxide, at a high ventilation rate, typically 30 × FEV1 (estimated 85% maximal voluntary ventilation) for 6–8 min [77]. The goal is to replicate the conditions of heavy breathing during exercise. The test is positive for EIB if it triggers bronchoconstriction and causes a FEV1 drop > or equal to 10% in lung function, measured using spirometry [77]. EVH is considered a reliable test for diagnosing EIB and is often used in clinical settings to evaluate individuals who experience exercise-related breathing difficulties. It also helps healthcare providers determine if bronchodilator medications or other treatments are needed to manage EIB. However, there are some limitations of the method, such as false positives and negatives, reproducibility, signs and symptoms mismatch, safety concerns, and not being suitable for all populations (i.e., cardiac diseases) [78]. Therefore, the conduct of EVH should be limited to well-trained experts, and strict adherence to safety measures is imperative. Several measurements are required to confirm or exclude EIB diagnosis [79]. Figure 2.

In a study that compared the treadmill test and EVH in asthmatic children, the agreement between the responses measured by the two bronchial challenge methods was moderate and the limits of agreement in FEV1 change as a percentage of basal values after the two challenges were very wide [80]. Therefore, the two tests are not interchangeable, and it is essential to thoroughly assess the consistency of the FEV1 response to the EVH challenge in order to gain a clearer understanding of its role in diagnosing EIB. EVH is recommended by the European Respiratory Society (ERS) and EAACI Task Force to identify EIBwa in athletes and it is included in the World Anti-Doping Agency assessment of asthma [17, 27, 81]. EVH is the preferred test for athletes suspected of having EIBwa, for the capacity to better replicate the conditions of sporting activity [82].

Furthermore, individuals with EIBwa typically exhibit normal FeNO levels, show a limited response to ICS, and in only a few cases, present with coexisting allergic conditions [82].

Nowadays, young athletes are commencing high-intensity training at an early age, emphasizing the importance of evaluation for EIB in this younger demographic. Thus far, no established screening test can enhance the initial likelihood of detecting EIB in young athletes. A step-by-step assessment would consist of a questionnaire based on clinical manifestations specifically on wheezing during exercise, atopic characteristics, spirometry before and after bronchodilator (to rule out EIBa), and additional markers in blood or serum. Initial screening tests can have the potential to identify (at a minimal expense) athletes at risk for EIB, which can subsequently be directed toward other tests [83].

There is no sport contraindicated for allergic children. It is important that the young patient engages in a sport he enjoys and manages the therapy for his underlying allergic condition appropriately. E.g., swimming, baseball, golf, martial arts, fencing, soccer, cycling, and volleyball are suitable options. For pediatric patients starting competitive sports, the potential risk of different sports should be discussed with the family [84].

## Treatment

The management of EIB in both athlete and non-athlete children should follow a similar approach, which includes avoiding environmental triggers, identifying and addressing any coexisting medical conditions, and focusing on the prevention and treatment of asthma. Sport is not contraindicated in children with EIB; on the contrary, regular physical exercise is recommended by international guidelines [8]. Physical activity plays a modulating role in lung inflammation, reducing bronchial hyperresponsiveness and the need for corticosteroids (inhaled or oral), as well as decreasing the number of eosinophils in sputum and FeNO levels [85, 86]. In a recent systematic review conducted by Silva et al., which encompassed articles assessing the potential of physical exercise to enhance the management and severity of EIB in children and adolescents, all the examined articles highlighted enhancements in cardiorespiratory fitness among the intervention group [87].

An effective training programme for children with asthma would consist of a personalised training intensity, a duration of at least three months, with at least two 60-minute training sessions/week [88].

Treatment of EIB includes pharmacological and non-pharmacological measures. Table 1.

Non-pharmacological measures to reduce or prevent EIB are inferior to pharmacological management but can be useful in many situations. Warming up is traditionally performed before any exercise: participating in a warm-up exercise can lead to the reduction of EIB in over half of the population. High-intensity interval or variable-intensity warm-up exercise before vigorous exercise, as opposed to no warm-up, leads to a statistically significant reduction in the percentage decrease in FEV1 [89]. This effect typically endures for 1–2 h, thereby minimizing the likelihood of bronchoconstriction during this refractory period. This temporary resistance is believed to occur due to the tachyphylaxis of airway smooth muscles to bronchoconstriction-inducing mediators [90]. Both continuous and intermittent warm-up exercises have been shown to reduce EIB [91, 92].

**Table 1:** Pharmacological and non-pharmacological treatments of EIBwa and EIBa

EIBwa	EIBa
**PHARMACOLOGIC INTERVENTIONS**: - SABA 5–20 min before exercise - Consider the addition of daily therapy with an inhaled corticosteroid, leukotriene receptor antagonist, or mast cell stabilizing agent before exercise, if clinical manifestations are not controlled with SABA.	**PHARMACOLOGIC INTERVENTIONS**: To achieve asthma control. Follow the international guidelines SABA 5–20 min before exercise
**NON-PHARMACOLOGIC INTERVENTIONS**: - Avoid activity in highly polluted areas and exposure to cold air - Perform a pre-exercise warm-up - Use a mask or scarf - Breathing through the nose	

It is standard practice to recommend the use of facemasks for the warming and humidification of inspired air in children with EIB during cold weather [6]. Breathing through the nose is preferable to maintain the optimal function of the upper airways, allowing for the humidification and warming of inspired air [17].

Pharmacological measures should follow treatment guidelines. In EIB, inhaled SABA are useful both as pre-treatment before competitions and to reduce signs and symptoms [6]. If SABA is not effective in preventing EIB, then addition of mast-cell stabilizing agents, such as cromolyn or nedocromil, or ICS, may be indicated [93, 94].

However, there were a considerable number of athletes with EIBwa who had less atopic predisposition and eosinophilic inflammation in whom an ICS was ineffective [95]. This so-called “sports asthma” syndrome is defined by the presence of exercise-induced respiratory symptoms and airway hyperresponsiveness in the absence of allergic features. The development of this pathophysiology is associated with specific training and environmental conditions, especially exposure to cold dry air and long exercise durations [96]. Genetic susceptibility, epithelial sensitivity, and neurogenic mechanisms should all be taken in account when explaining the athlete’sindividual risk of bronchoconstriction [97]. The phenotype that involves less eosinophilic inflammation should be individualized and properly treated [82].

Optimal control of EIBa should be obtained first treating properly asthma disease according to international recommendations [7]. This treatment includes many drugs, but is based on anti-inflammatory treatment through inhaled corticosteroids (ICS). The dose may vary according to asthma severity, but the effect occurs quickly, beginning after one week of treatment and improving further through the following weeks. The results of many studies indicate that ICSs diminish the severity of EIB in all individuals with asthma [74, 81, 97].

Antiasthmatic drugs can be assumed by athletes, according to regulatory agencies.

The last version of the World Anti-Doping Code (WADA) includes, in the list of prohibited substances for athletes, all selective and non-selective beta-2 agonists, including salbutamol, formoterol and salmeterol. However, inhaled salbutamol can be used for a maximum of 1600 micrograms over 24 h in divided doses not exceeding 600 micrograms over 8 h starting from any dose; inhaled formoterol can be taken for a maximum delivered dose of 54 micrograms over 24 h; inhaled salmeterol can be used for maximum 200 micrograms over 24 h. Inhaled administration of glucocorticoids is not prohibited when used within the manufacturer’s licensed doses and therapeutic indications [98].

## Conclusion

In conclusion, EIB is common in pediatric age and should be recognized and properly treated through non-pharmacological and pharmacological measures. SABA and, in some cases, mast cell stabilizers and ICS are usually efficacious in the control of signs and symptoms. In EIBa, the optimal control of asthma reduces the frequency and intensity of EIB. Concomitant allergic diseases should be considered a risk factor of EIB and should be properly treated. EIB in athletes needs an individualized approach.

## Scenarios

### A) A child with allergic rhinitis who wants to engage in competitive sports

F. is a sportive 10-year-old girl suffering from allergic rhinitis. No history of asthma. She was always actively involved in playing football and, this year, she intended to pursue it at a competitive level. She, along with her parents, seek guidance on effectively managing her condition to ensure it did not deteriorate or hinder her participation in more advanced sports activities. SPT were positive for *Dermatophagoides Pteronissinus* and grass. Spirometry before and after the bronchodilatation test and ACT were normal.

Antigen avoidance, adequate daily nasal hygiene, a second-generation H1- antihistamine and, if necessary, topical nasal corticosteroids were recommended. Furthermore, allergen-specific immunotherapy may be added to reduce the need and amount of additional medication. Periodic follow-ups are established to monitor signs and symptom control and assess the emergence of EIB or other respiratory signs.

**Comment**: about 20–40% of children with rhinitis have EIB, particularly those with persistent untreated clinical manifestations. Treatment of the allergic condition and periodic follow-ups arerecommended.

### B) A child with intermittent wheezing and sensitization to dust mites experiencing EIB

A. is a 9-year-old child with a history of allergic rhinitis, intermittent wheezing and sensitisation to dust mites. He is in treatment with low-dose ICS taken whenever SABA was taken. Their parents report a worsening of signs and symptoms with the occasional appearance of dyspnea during exercise. The chest auscultation and the spirometry test with bronchodilation were normal. The next day he performs the exercise challenge test, which reveals a FEV_1_ fall of 20% after 15 min. He receives the diagnosis of EIB with asthma, and low doses of ICS and administration of inhaled SABA before exercise was recommended. After 3 months, A. had good asthma control and no longer experienced episodes of shortness of breath during physical exercise. Periodic follow-ups to monitor signs and symptoms control and assess spirometry and ECT were scheduled.

**Comment**: EIB is frequently associated with asthma, and both asthma and EIB should be properly diagnosed and treated.

### C) An adolescent athlete experiencing EIB

V. is a 15-year-old athlete swimmer who experiences “shortness of breath” during the long duration training sessions. He has no history of allergic rhinitis, atopic dermatitis or asthma. Spirometry and BD test is normal and SPTs are negative. He performs an EVH test that results positive for EIB.

Proper warm-up and SABA inhalation 5–20 min before planned exercise are recommended. Despite the use of SABA, the patient still suffered from EIB signs and symptoms. Thus, the addiction of daily monotherapy with ICS, is prescribed. In subsequent follow-ups, he no longer reports the occurrence of respiratory clinical manifestations during exercise.

**Comment**: Many adolescents practice high-intensity training. EIBwa is particularly frequent in athletes, who need an individualized approach.

## Data Availability

Not applicable.

## Abbreviations

AAAAI: American Academy of Allergy, Asthma & Immunology
ACAAI: American College of Allergy, Asthma & Immunology
ARIA: Allergic Rhinitis and its Impact on Asthma
EAACI: European Academy of Allergy and Clinical Immunology
EIA: Exercise-induced asthma
EIB: Exercise-induced bronchoconstriction
EIBa: EIB with asthma
EIBwa: EIB without asthma
ERS: European Respiratory Society
GINA: Global Initiative on Asthma
ICS: Inhaled corticosteroids
JCAAI: Joint Council of Allergy Asthma & Immunology
JTFPP: Joint Task Force on Practice Parameters
LABA: Long-acting b2-agonists
PERF: Peak Expiratory Flow Rate
SABA: Short-acting b2-agonists
WADA: World Anti-Doping Code
